# Infective endocarditis in developing countries: An update

**DOI:** 10.3389/fcvm.2022.1007118

**Published:** 2022-09-12

**Authors:** Reuben K. Mutagaywa, Josephine C. Vroon, Lulu Fundikira, Anna Maria Wind, Peter Kunambi, Joel Manyahi, Apollinary Kamuhabwa, Gideon Kwesigabo, Steven A. J. Chamuleau, Maarten J. Cramer, Pilly Chillo

**Affiliations:** ^1^Division of Heart and Lung, Department of Cardiology, Faculty of Medicine, University Medical Centre Utrecht, Utrecht, Netherlands; ^2^Department of Internal Medicine, School of Medicine, Muhimbili University of Health and Allied Sciences, Dar es Salaam, Tanzania; ^3^Department of Cardiology, Diakonessen Hospital, Utrecht, Netherlands; ^4^Department of Clinical Pharmacy and Pharmacology, School of Pharmacy, Muhimbili University of Health and Allied Sciences, Dar es Salaam, Tanzania; ^5^Department of Epidemiology and Biostatistics, School of Public Health, Muhimbili University of Health and Allied Sciences, Dar es Salaam, Tanzania; ^6^Amsterdam UMC Heart Center, Department of Cardiology, Amsterdam Cardiovascular Sciences, Amsterdam University Medical Centre, Amsterdam, Netherlands

**Keywords:** infective endocarditis, morbidity, mortality, developing countries, rheumatic heart disease

## Abstract

**Introduction:**

Despite advances in diagnostic and treatment, morbidity and mortality due to infective endocarditis (IE) has not decreased. There is a discrepancy in epidemiology of IE between developed and developing countries. Over the last years, increased early detection and consequently prevalence of rheumatic heart disease (RHD) and congenital heart disease (CHD) which are considered predisposing conditions for IE, is noted. Here, we present a review of literature on IE in developing countries.

**Methods:**

We conducted a systematic literature search of IE studies in developing countries through PubMed and Embase. We have divided the studies into two groups: studies published before 2015 (group 1) and studies ≥ 2015 (group 2). The outcome was defined as a difference in epidemiology, microbiology, treatment, and mortality over time. The Scale for Assessment of Narrative Review Articles guidelines was applied.

**Findings:**

In total, 16 studies were included. The total number of IE cases was 1,098 and 1,505 in groups 1 and 2, respectively. We compared 4/7 cohorts from group 1 (*n* = 789) with 5/9 cohorts from group 2 (*n* = 636). Six studies were not included in the comparison because they were interacting between the two cohorts. Males predominated in all studies. Rheumatic heart disease was higher in group 1 than in group 2 (42.3% vs. 30.3%, *p* < 0.001) while for CHD there was no change (17.6% vs. 16.7%, *p* = 0.672). Streptococci infections was lower in group 1 than group 2 (26.2% vs. 37.7%, *p* < 0.001). The proportion of *Staphylococcus aureus* was 15.3% in group 1 and 23.6% in group 2, *p* < 0.001. Negative blood culture (NBC) was higher in group 1 than in group 2 (42.2% vs. 34.1%, *p* = 0.002). Patients in group 1 received more surgery than in group 2 (38.8% vs. 28.8%, *p* < 0.001). Mortality was similar in the two groups (20.9% vs. 22.3%, *p* = 0.518).

**Conclusion:**

This review shows a scarcity of studies on IE in developing countries. Rheumatic heart disease and congenital heart disease are common predisposing conditions. Other risk factors are prosthetic valves, degenerative valve disease (DVD), intravenous drug use, and human immunodeficiency virus infection. While the proportion of IE cases caused by *Streptococcus* and *Staphylococcus* has increased, the number of NBC and patients getting surgery has decreased. Mortality has not changed over time. Timely diagnosis and management of patients with RHD and CHD and comprehensive management of IE are warranted.

## Introduction

Infective endocarditis (IE) is a complex disease associated with a burden on the healthcare system due to its imposing prolonged hospitalization, a high mortality rate of about 20–25%, and high morbidity (1–4). Worldwide the incidence of IE is still rising, despite the improvement in diagnostics and treatment options. The risk factors for developing IE have been evolving over the last decade (5). In high-income countries (HIC), advances in interventional cardiology with devices came at the cost of increased device-related infections. Prosthetic valve endocarditis now accounts for approximately 20% of all endocarditis cases in HIC (6). Advances in diagnostic tools like positron emission tomography (PET) scan and transesophageal echocardiography (TEE), availability of modern treatment options, and the creation of “endocarditis team” have improved the management and outcomes of IE in HIC (7–11).

To the best of our knowledge, little is known about IE in developing countries due to a lack of adequate studies in this area. Rheumatic heart disease (RHD) and congenital heart disease (CHD) have been reported as the most common cause of IE (3, 12). Studies have shown that, in developing countries, optimal medical and surgical management is limited (12). In these countries, microorganisms are reported to be unidentified due to poor diagnostic capacity (3). Moreover, studies have shown that patients have more complications due to delays in diagnosis and late hospital presentation (3, 12).

Recently, in developing countries, there has been an increase in the use of prosthetic and intra-cardiac devices which are other risk factors of IE (12). Patients who are at risk of developing IE also include those who visit the healthcare system for other comorbidities, for example, immunosuppressed patients (1, 5). The microbiological spectrum of IE has also been changing field (3, 12). Some studies report that *Staphylococcus aureus* is now the most common organism, especially due to the healthcare-associated IE (3, 12–14). However, other studies show that streptococci still predominates (15, 16). Furthermore, the proportion of cases of IE that are caused by coagulase-negative staphylococci (CoNS) are rising concurrently with the decrease of *Streptococcus viridians* and *Enterococcus* (3, 12).

In the present study, we are summarizing the current state of IE in developing countries and to investigate whether there is a change over time in the presentation of IE. We have assembled information about the epidemiology, diagnosis, treatment, and mortality of IE in these developing countries. Our review includes publications of studies from the year 2000 to 2020 with two predefined groups: studies published before the year 2015 (group 1, the “early” cohorts) and the year ≥ 2015 (group 2, the “late” cohorts). In the end, we have highlighted the future perspectives toward comprehensive management of IE in developing countries.

## Methods

### Search strategy

A systematic literature search was performed through PubMed and Embase using the keywords “endocarditis,” “developing country,” “poverty,” and “low- and middle-income country.” The identified records were entered in Rayyan QRCI and were independently screened by two blinded reviewers (JV and RM). Subsequently, a full-text review of the remaining studies was performed, and studies were selected if eligibility criteria were fulfilled. Disagreements were resolved by consensus. The detailed search queries are Pubmed: *(((endocarditis [MeSH Terms]) OR “endocarditis”[Title/Abstract])) AND (((((developing countries [MeSH Terms]) OR developing country [MeSH Terms]) OR low-income population [MeSH Terms]) OR “developing countries”[Title/Abstract]) OR “low income countries”[Title/Abstract])* and Embase: '*endocarditis': ti, ab, kw AND ('developing country': ti, ab, kw OR 'low income country': ti, ab, kw OR 'low middle income country': ti, ab, kw OR 'lowest income group': ti, ab, kw)*.

### Study eligibility and definitions

Any study which reported detailed information about the IE population from developing countries was considered eligible for this research. The inclusion criteria were English language and publication date from the year 2000–2020. Exclusion criteria comprised studies that entirely included children only and studies in which full text was not available. Developing countries were defined according to the International Monetary Fund definition (17, 18). Our study population was defined as patients diagnosed with possible or definite endocarditis by using the revised Duke criteria (19). All studies had to report on the number of IE subjects included in that particular study, age, and sex of the study population and it was required to describe data on numbers of antibiotic and surgical treatment and (in-hospital) mortality. Besides, data concerning predisposing conditions and microbiology was mandatory. For the sake of determining the trends in different parameters concerning IE and for comparison purposes, we have divided our study population into two groups namely studies published before the year 2015 (group 1, “early” cohorts) and studies published in the year ≥ 2015 (group 2, “late” cohorts). “Early” cohorts are studies that entirely recruited patients from the year 1986–2005 while “late” cohorts are studies that entirely recruited patients from the year ≥ 2005–2017. The reporting of this study conforms to SANRA (the Scale for Assessment of Narrative Review Articles) guidelines (20).

### Data analysis

Descriptive summaries of the data are presented. Continuous parameters are reported as mean and standard deviation or median and interquartile range. Discrete variables are presented in percentages. The Chi-square and Fisher's exact tests were used to compare categorical data. SPSS (v.28) was used for analysis. *P*-value < 0.05 was considered statistically significant.

## Results

**Figure 1:**
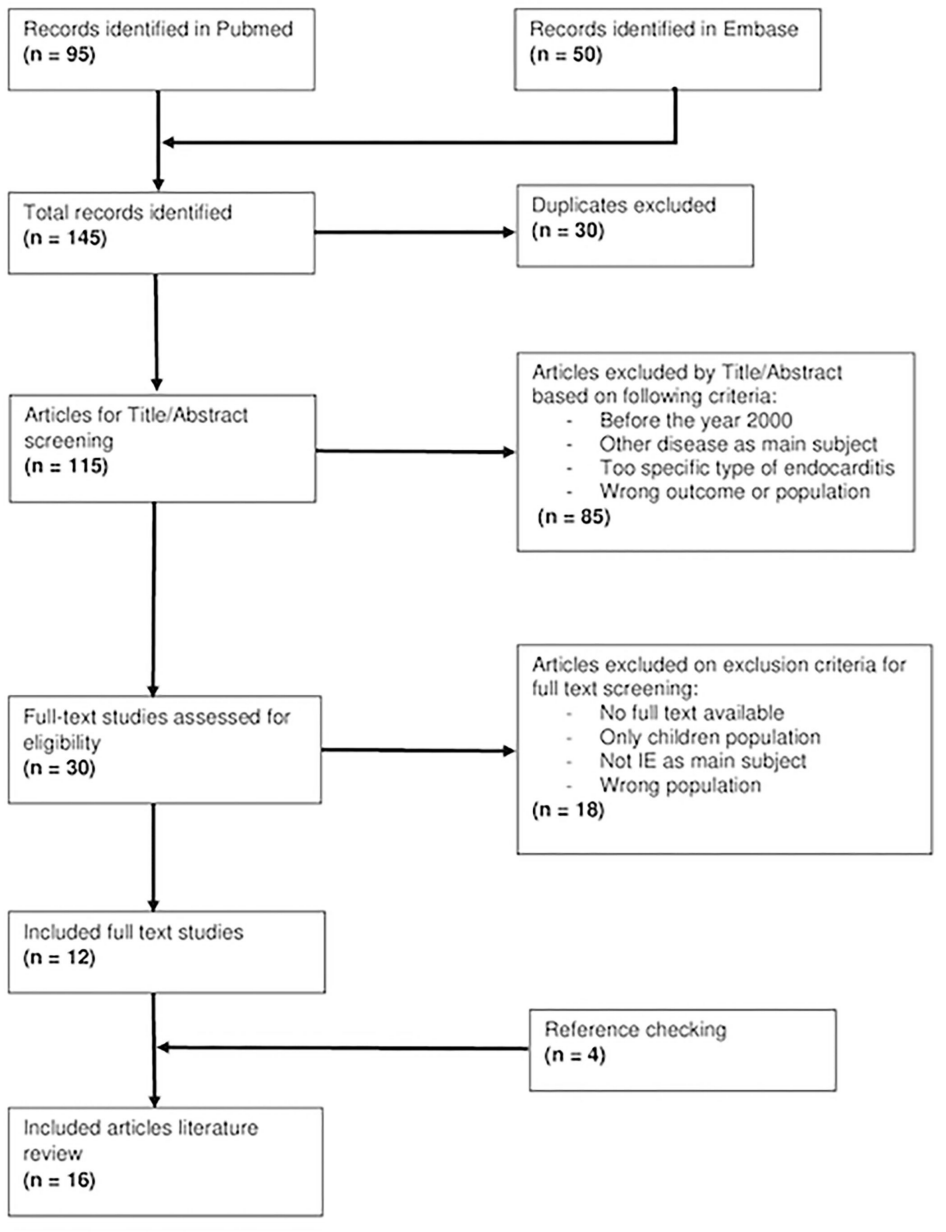
A flowchart showing a literature search of included studies.

Our search resulted in 145 articles of which 12 studies eventually are included. All of the included studies were Google searched and cross-referenced for an additional of four relevant articles which brought a total of sixteen studies from nine countries (Figure 1).

### Study characteristics and demographics

All study characteristics are shown in Tables 1, 2. We retrieved seven studies for group 1 and nine studies for group 2. In the first group, the total number of IE cases was 1,098 whereas in the second group it was 1,505. The lowest mean age was 23.5 (interquartile range, 9–38) years while the highest was 59 ± 17.8 years. In all studies except one, there were more males than females. The clinical case definition used for diagnosing IE was reported in 12 (75%) studies of which 100% of definite diagnosis was only in three studies.

**Table 1:** Characteristics of the study population of group 1, publications before 2015.

**Year**	**Author**	**Cohort**	**Country**	**Study design**	**IE** **cases**	**Definite vs. Probable (%)**	**Native vs. PHV (%)**	**Mean age ±SD (years)**	**Male** **(%)**	**Predisposing condition (%)**	**Microbiology (%)**	**Antibiotics (%)**	**Surgery (%)**	**In-hospital mortality (%)**
2002	Kanafani et al. (21)	1986–2001	Lebanon	Retrospective	91	82 vs. 17	80 vs. 20	48 ± 19	64	RHD (33) CHD (13) DVD (NR) IVD (0) HIV (0)	NBC (23) *Strept* (51) *S. viridans* (28) *Staph* spp. (36) *S. aureus* (26) Enteroc (4) CoNS (10)	100	32	18
2004	Tariq et al. (22)	1997–2001	Pakistan		66	50 vs. 50	92 vs. 8	Mean 28.8 Median 24	60	RHD (23) CHD (50) DVD (2) IVDA (NR) HIV (NR)	NBC (48) *Strept* (59) *Staph* spp. (24) Enteroc (3) Gramneg (15)	100	12	27
2005	Garg et al. (23)	1992–2001	India	Retrospective	192	100 vs. 0	90 vs. 10	27.6 ± 12.7	73	RHD (47) CHD (29) DVD (3) IVD (NR) HIV (NR)	NBC (32) *Strept* (23) *Staph* spp. (20) *S. aureus* (15) CoNS (5) Enteroc (8) Gramneg (13)	100	23	21
2007	Letaief et al. (24)	1991–2000	Tunisia	Retrospective	440	NR	83 vs. 17	32.4 ± 16.8	56	RHD (45) CHD (9) DVD (6) IVDA (0) HIV (NR)	NBC (50) *Strept* (18) *Staph* spp. (18) *S. aureus* (12) CoNS (6) Enteroc (4)	100	51	21
2008	Trabelsi et al. (25)	1997–2006	Tunisia	Retrospective	134	93 vs. 7	100 vs. 0	Mean 34.2 (IQR 4–73)	58	RHD (45) CHD (16) DVD (8) IVDA (NR) HIV (NR)	NBC (49) *Strept* (24) *Staph* spp. (22) *S. aureus* (18) CoNS (4) Bartonella (8) Enteroc (1)	100	51	19
2011	Math et al. (16)	2004–2006	New Delhi, India	Prospective	104	100 vs. 0	30 vs. 20	Mean 23.5 (IQR 9–38)	71	RF (5) CHD (39) IVDA (0) HIV (NR)	NBC (59) *Strep*t (8) *S. aureus* (7) Enteroc (5)	100	15	26
2014	Damasco et al. (13)	2009–2013	Brazil	Retrospective	71	79 vs. 21	90 vs. 10	49.8 **±** 2.4	58	NR	NBC (15.5) *Strept* (25) *S. aureus* (30) Enteroc (27) CoNS (8)	NR	NR	46

RHD, rheumatic heart disease; CHD, congenital heart disease; DVD, degenerative valve disease; RF, rheumatic fever; NR, not reported; PHV, prosthetic heart valve; IE, infective endocarditis; NBC, negative blood culture; CoNS, coagulase negative staphylococci; IVDA, intravenous drug abuse; HIV, human immunodeficiency virus; Staph spp., Staphylococcus spp., Strept, Streptococcus spp., enteroc, enterococci; Gramneg, Gram negative.

**Table 2:** Characteristics of the study population of group 2, publication ≥2015 to 2019.

**Year**	**Author**	**Cohort**	**Country**	**Study design**	**IE cases**	**Definite vs. probable (%)**	**Native vs. PHV (%)**	**Mean age ±SD (years)**	**Male** **(%)**	**Predisposing condition (%)**	**Microbiology (%)**	**Antibiotics (%)**	**Surgery (%)**	**In-hospital mortality (%)**
2015	Mirabel et al. (15)	2006–2012	Lao PDR	Retrospective	36	31 vs. 69	83 vs. 17	25 (IQR 18–42)	42	RHD (33) CHD (19) DVD (8) IVDA (NR) HIV (NR)	NBC (61) *Strept* (19) *Staph* spp. (6) *E. coli* (6) Enteroc (6) *S. aureus (3)* CoNS (3)	69	0	39
2016	Xu et al. (26)	2008–2011	East China	Retrospective	66	73 vs. 27	96 vs. 4	46.3 **±** 16.1	61	RHD (33) CHD (19) DVD (8) IVDA (1)	NBC (39) *Strept* (56) *Staph* spp. (26) Enteroc (3)	100	32	10
		2012–2015			108	81 vs. 19	93 vs. 7	48.7 ± 15.5	69	RHD (27) CHD (12) DVD (33) IVDA (0)	NBC (41) *Strept* (65) *Staph* spp. (21) Enteroc (6)	100	51	12
2017	Fernandes et al. (27)	2000–2012	West Indies	Retrospective	201	100 vs. 0	90 vs. 10	Median 48	67	BHD (54) CHD (NR) DVD (NR) IVDA (2) HIV (27)	NBC (21) *Strept* (30) *Staph* spp. (29) *S. aureus* (23) CoNS (6) Enteroc (5)	100	53	19
2017	El-chakhtoura (28)	1989–2001	Lebanon	Retrospective	86	80 vs. 20	80 vs. 20	48 ± 18.2	62	RHD (15) CHD (8) DVD (NR) IVDA (0) HIV (0)	NBC (26) *Strept* (40) *Staph* spp. (26) *S. aureus* (20) CoNS (6) Enteroc (4)	NR	33	15
		2001–2014			80	80 vs. 20	70 vs. 30	59 **±** 17.8	75	RHD (16) CHD (9) DVD (NR) IVD (1) HIV (1)	NBC (16) *Strept* (26) *Staph* spp. (31) *S. aureus* (20) CoNS (11) Enteroc (15)	NR	31	16
2017	Tran et al. (29)	2005–2014	Vietnam	Retrospective	189	NR	87 vs. 12	38 **±** 18	64	VHD (66) CHD (19) DVD (NR) IVDA (1) HIV (NR)	NBC (30) *Strept* (75) *Staph* spp. (10) Gramneg (5) Enteroce (4)	NR	NR	7
2018	Subbaraju et al. (30)	2007–2013	South India	Retrospective	139	68 vs. 32	96 vs. 4	47.9 **±** 15.8	68	RHD (31) CHD (16) DVD (23) IVDA (0)	NBC (30) *S. pyogen* (31) *S. aureus* (11) Enteroc (13)	100	4	17
2019	Ren et al. (31)	2001–2009	South China	Retrospective	97	NR	97 vs. 3	36.5 **±** 15.2	72	BHD (49) CHD (21) RHD (23) DVD (4) IVDA (26)	NBC (53) *Strept* (37) *S. aureus* (41) Enteroc (2)	100	59	13
		2010–2018			216	NR		44.9 **±** 15.4	72	BHD (44) CHD (15) RHD (18) DVD (9)	NBC (38) *Strept* (44) *S. aureus* (20) Enteroc (10)	100	60	10
2019	Villiers et al. (14)	2009–2016	Cape Town	Retrospective	105	65 vs. 35	84 vs. 16	Median 39 (IQR 29–51)	62	RHD (34) CHD (11) IVDA (14) HIV (23)	NBC (41) *Strept* (17) *S. aureus* (19) Enteroc (7)	100	42	19
2019	Sunil et al. (32)	2005–2017	Malaysia	Retrospective	182	84 vs. 16	94 vs. 6	50.0 **±** 17.6	70	RHD (42) CHD (8) DVD (NR) IVD (5) HIV (3)	NBC (22) *Strept* (36) *S. viridans* (29) *S. aureus* (41) Enteroc (9)	100	32	37

RHD, rheumatic heart disease; CHD, congenital heart disease; BHD, basic heart disease; DVD, degenerative valve disease; VHD, valvular heart disease; NR, not reported; PHV, prosthetic heart valve; IE, infective endocarditis; NBC, negative blood culture; IVDA, intravenous drug abuse; HIV, human immunodeficiency virus; Staph spp., Staphylococcus spp.; Strept, Streptococcus spp.; S. pyogen, S. pyogenes; enteroc, enterococci.

We performed a detailed comparison of four studies [cohorts by Kanafani et al. (21), Tariq et al. (22), Garg et al. (23), and Letaief et al. (24)] from group 1 with five cohorts [by Mirabel et al. (15), Xu et al. (26), Subbaraju et al. (30), Villers et al. (14), and Sunil et al. (32)] from group 2 for several parameters. The remaining cohorts were either interacting between the two groups or had missing data and therefore they were not considered for comparison but were described accordingly. The prevalence of males was lower in group 1 than in group 2 (62.0% vs. 65.6%, *p* = 0.167) as shown in Table 3. The proportion of native valves affection was lower in group 1 than in group 2 (84.9% vs. 90.3%, *p* = 0.003). The proportion of IE on prosthetic valves was higher in group 1 than in group 2 (15.1% vs. 7.4%, *p* < 0.001) as depicted in Table 3.

**Table 3:** Comparison of the two IE cohorts in respect to several parameters.

	**<2005 (*N* = 789)**	**≥2005 (*N* = 636)**		
**Variable**	***n* (%)**	***n* (%)**	**Difference (95% CI)**	***P*-value**
Sex (Male)	489 (62.0)	417 (65.6)	−0.04 (−0.09, 0.01)	0.167
Native	670 (84.9)	574 (90.3)	−0.05 (−0.09, −0.02)	0.003
Prosthetic	119 (15.1)	47 (7.4)	0.08 (0.04, 0.11)	<0.001
RHD	334 (42.3)	193 (30.3)	0.12 (0.07, 0.17)	<0.001
CHD	139 (17.6)	106 (16.7)	0.01 (−0.03, 0.05)	0.672
*Streptococcus*	207 (26.2)	240 (37.7)	−0.12 (−0.16, −0.07)	<0.001
*Staphylococcus*	121 (15.3)	150 (23.6)	−0.08 (−0.12, 0.04)	<0.001
Culture negative	333 (42.2)	217 (34.1)	0.08 (0.03, 0.13)	0.002
Surgery	306 (38.8)	183 (28.8)	0.10 (0.05, 0.15)	<0.001
Mortality	165 (20.9)	142 (22.3)	−0.01 (−0.06, 0.03)	0.518

### Predisposing cardiac conditions

**Figure 2:**
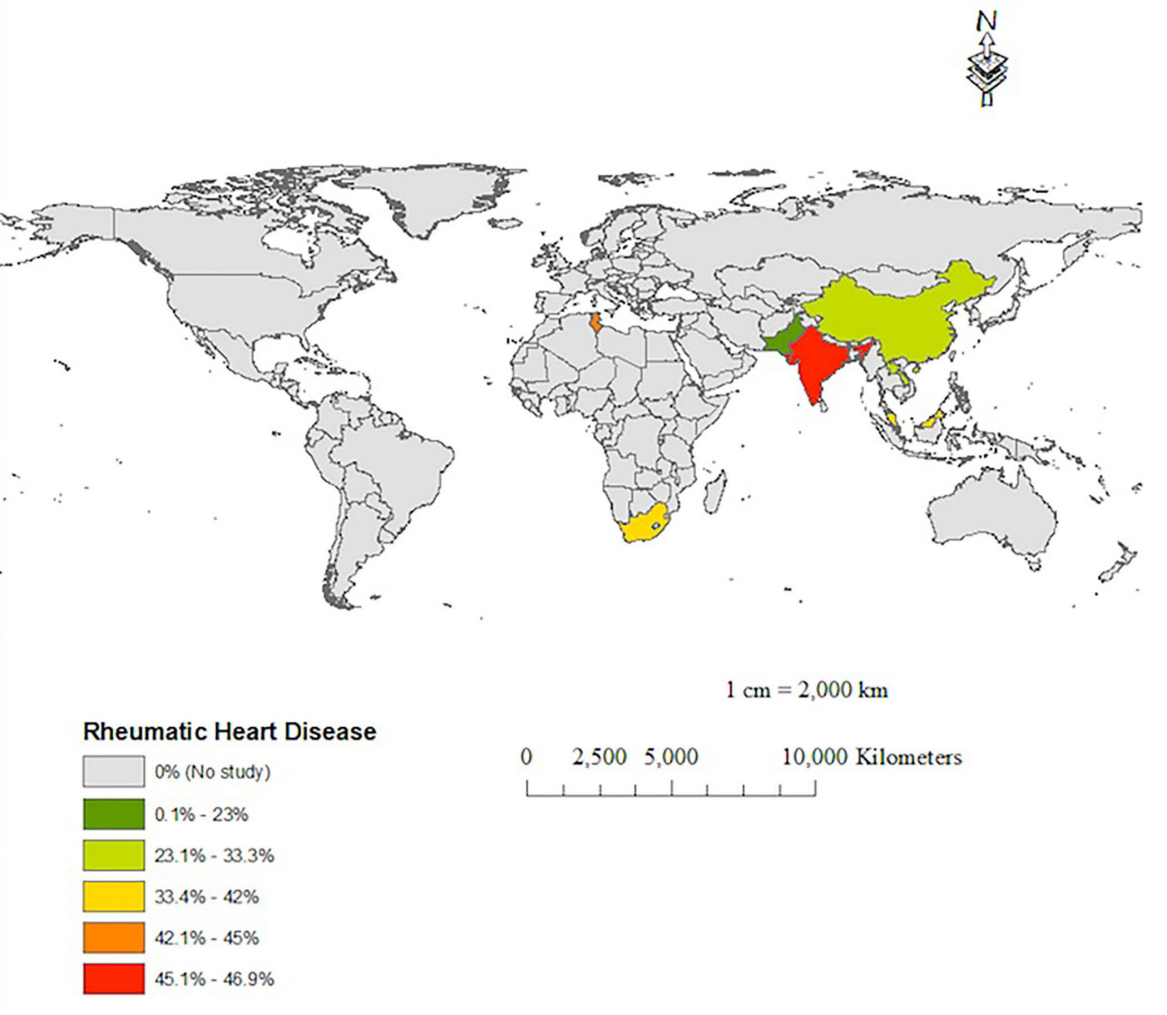
A map showing the prevalence of rheumatic heart disease as a predisposing condition of IE in the reported IE studies.

Tables 1, 2 show a wide range of rheumatic (23–46.9%) and CHD (7.7–50%), the two conditions were reported in 13 (18.25%) of the reviewed studies. As shown in Table 3, the prevalence of RHD was significantly higher in group 1 than in group 2 (42.3% vs. 30.3%, 0 < 0.001). Congenital heart disease occurred with a similar magnitude between groups 1 and 2 (17.6% vs. 16.7%, *p* = 0.672). Tables 1, 2 depict that the prevalence of degenerative valve disease (DVD) was ranging from 4% to 33%, being reported in half of the reviewed studies. The proportion of intravenous drug use and HIV infections were ranging from (0–25.8%) to (0–27%), respectively, but they were rarely reported as shown in Tables 1, 2. Overall, there was under-representation of Africa where many developing countries belongs. Figure 2 exemplifies countries that were recruited in this review with a reference to the prevalence of RHD as the common predisposing cardiac condition of IE.

### Microbiology

As depicted in Table 3, the proportion of Streptococci spp. was lower in group 1 than in group 2 (26.2% vs. 37.7%, *p* < 0.001) with the common isolates being *Streptococcus pyogenes* and *Streptococcus viridans*. Similarly, the presence of Staphylococci spp. in the early cohorts was less common than in the late cohorts (15.3% vs. 23.6%, *p* < 0.001) with the common isolates being *S. aureus*. The proportion of coagulase-negative *Staphylococcus* occurred in the range of 2.8–11.3% while that of enterococci was ranging from 1% to 26.7% (Tables 1, 2). The proportion of negative blood culture (NBC) was significantly higher in group 1 than in group 2 (42.2% vs. 34.1%, *p* = 0.002) as shown in Table 3.

### Medical treatment

As shown in Tables 1, 2, antibiotics were prescribed by 100% in all of the reported studies except one. Penicillin with or without aminoglycosides was the most used antibiotic across all cohorts. Of the aminoglycosides, gentamicin was the most commonly used antibiotic. Ceftriaxone and vancomycin were also regularly used. In group 1, Letaief et al. (24) and Trabelsi et al. (25) did not report on the antibiotic used and only Math et al. (16) mentioned larger combinations of therapy in addition to fluoroquinolones. In group 2, Xu et al. (26) often used glycopeptides and cephalosporin, Subbaraju et al. (30) mostly used gentamicin and ceftriaxone, and Sunil et al. (32) most frequently used ceftriaxone, with or without benzylpenicillin. Fernandes et al. (27), Tran et al. (29), Ren et al. (31), and Villiers et al. (14) did not report on the kind of antibiotics used.

### Surgery

Fifteen (93.8%) of 16 studies reported surgical treatment of IE. The rate of surgery performed varied across the reviewed studies from 0% in Lao to 60.2% in China (Tables 1, 2). Among the compared groups, the proportion of patients who underwent surgery was higher in group 1 than in group 2 (38.8% vs. 28.8%, *p* < 0.001) as shown in Table 3. Damasco et al. (13) and Tran et al. (29) did not report on surgical intervention. In a report by Mirabel et al. (15), no surgical intervention was offered to patients with IE in Lao People's Democratic Republic.

### Mortality

Mortality data were reported in all of the studies (Tables 1, 2). As shown in Table 3, the in-hospital mortality did not significantly differ between group 1 and group 2 (20.9% vs. 22.3%, *p* = 0.518). This signifies that mortality as a result of IE has not changed over the past four decades of the reviewed cohorts i.e. 1986–2017.

## Discussion

The epidemiology of IE in developing countries has been reported in a few studies some of which give inconsistent results (3, 12). In the past, the epidemiology of IE in developing countries was reported to be similar to that of HIC (3, 4). Anecdotal evidence shows that in developing countries IE is not an uncommon condition although some hospital records report that IE accounts for <0.5% of admissions due to cardiovascular conditions (33, 34). It is challenging to make a diagnosis of IE in areas where causes of fever are plenty and therefore high expertise in clinical suspicion complemented by appropriate laboratory investigations is needed. This is further hampered by the poor health infrastructures and patients' financial constraints in which most of these patients do not own health insurance. This review was undertaken to summarize the current state of IE in developing countries and to investigate whether there is a change over time in the presentation of IE. The review had a main focus on epidemiology, diagnosis, treatment, and mortality of IE in these developing countries. However, suffice it to say we know little about IE in most parts of sub-Saharan Africa.

In this review, the lowest mean age of patients was 23.5 (interquartile range, 9–38) years while the highest was 59 ± 17.8 years. This is similar to the mean age of 47 years reported by Njuguna et al. (3) and the mean age of fewer than 40 years in a systematic review done by Noubiap et al. (12). These findings imply that IE in developing countries affects the young and this is probably due to the common predisposing conditions in these areas which are RHD and CHD. On the contrary, native valve IE in the HIC commonly affects the old population of which DVD is the major underlying cardiac disease (6, 35, 36). Our review showed a male predominance in all except one study. Similarly, previous studies have reported a high prevalence of males in IE studies (3, 12). The reason for the increased proportions of males in these studies has not been elucidated, although estrogen is implicated in protection against endothelia damage (37).

This review showed that the proportion of patients with native valve IE was significantly lower in group 1 (84.9%) than in group 2 (90.3%). The higher proportion of patients with native valve IE in the second cohort could be explained by the fact that awareness and diagnostic tests have probably increased in the latest decades hence increasing the detection rate. Similarly, a recent systematic review has reported that Native valves were involved in 81.1% of patients with IE (12). The same observation has been reported in a study from HIC in which native valve IE accounted for 72% (6). On the other hand, the proportion of patients with prosthetic valve IE was significantly higher in group 1 (15.1%) than in group 2 (7.4%). This could probably be due to improved care of patients with prosthetic valves in recent decades. In contrast, Noubiap et al. (12) reported that 18.2% of patients with IE had prosthetic valves. The authors argued that this could be due to increased access to cardiac surgery and/or a reporting bias because these patients are likely to receive regular medical follow-up with subsequent early detection of IE if occurs. On the other hand, native valve IE commonly go undiagnosed until when it has resulted into complications.

### Predisposing conditions

The current review showed that RHD was the leading predisposing condition for IE followed by CHD. Similarly, previous studies have reported the same findings (3, 12), implying the endemicity of RHD in developing countries. In contrast, in HIC, RHD accounts for 3% of patients with IE (6). However, with migration, RHD is evolving in the HIC (38) and hence the prevalence of IE in HIC may also increase. The prevalence of RHD as a predisposing condition for IE was statistically significantly higher (42.3%) in the first group than in the second group (30.3%). The reasons for a decrease in the prevalence of RHD as a predisposing condition for IE in group 2 as compared to group 1 could probably, among other reasons, be due to improved hygiene and the use of prophylactic antibiotics when patients are undergoing risky procedures. The prevalence of CHD as a predisposing condition of IE in group 1 and group 2 was similar (17.6% vs. 16.7%, *p* = 0.672). The reason for the similarity in the proportion of CHD in the two groups is possible because no interventions have been provided over time given the fact that adult CHD are less likely to be predisposed to IE. In contrast, a recent review of previous studies which were mostly performed in adults have reported that IE occurred in only 8% of patients with CHD (12). The observed differences between the two reviews are due to the fact that some of our reviewed studies comprised children. Congenital heart disease is a common risk factor for IE in children accounting for about 50% in several studies (3, 12).

Our review showed that DVD was rare in patients with IE, this is contrary to findings from HIC where it is the commonest predisposing cardiac condition in native valve IE (6). The reason for this observation is probably because in developing countries IE affects the younger patients as was seen in our reviewed studies. Similarly, both intravenous drug use and human immunodeficiency virus were rare predisposing conditions for IE. However, our findings should be interpreted with caution because most of the reviewed studies did not report on these conditions. Indeed, several reports have shown that intravenous drug use is not uncommon in developing countries (12, 39), but lower than what has been reported in HIC (40). Moreover, several studies have reported human immunodeficiency virus as a predisposing factor for HIV-associated cardiac disease including IE (13, 14, 41).

### Microbiology

It is important to identify the causative microorganisms of IE to offer targeted antimicrobial therapy. Unfortunately, in our review nearly half of the patients with IE in group 1 no microorganism was detected, opposing to HIC where microorganisms are identified in 95% of cases (4). Possible explanation for such a low detection rate observed in our review could be due to the use of antibiotics before the collection of blood, poor infrastructures for laboratory tests, and unavailability of standard operating procedures for blood collection and processing (3, 4, 12). Due to financial constraints, it is likely that very few blood cultures are done in developing countries (32). This in turn, has a consequence in the overall management of IE. Our review showed a decrease in the number of NBC in the late cohorts probably be due to the overall improvement in the standard of health care observed over time. Failure to do blood culture in patients with IE in developing countries is a concern when one suspect fastidious organism. With the use of newer blood culture techniques (mass spectrometry) which allow direct detection of bacterial species, the incidence of NBC IE may drop significantly (42).

Our review revealed that *Streptococcus* spp. continues to be the leading cause of IE, followed by *Staphylococci* spp. and that both of the two species have increased over time. Our findings are similar to what has been reported in previous studies (3, 43). On the contrary, Noubiap et al. (12) has reported in their review that *Staphylococcus* is the leading cause of IE in Africa, same as it is in HIC (6, 36). The difference observed between our review and that of Noubiap et al. could be due to the difference in the studied populations, we recruited studies across many developing countries and mostly adults while they reviewed African studies with a large proportion of children. The observation that both of the two genera have increased over the compared two time period, is a concern. Infective endocarditis due to *Staphylococcus* is relatively fatal and is associated with antimicrobial resistance, recently methicillin resistance *Staphylococcus aureus* (MRSA) has been reported to be a global health problem (4). This scenario is particularly important in developing countries where susceptibility tests are not routinely done. Of the common *Streptococcus* spp. reported in our review were *S. pyogenes* and *S. viridans* similar to what has been reported in previous studies (3, 12). It is worth to mention that *S. pyogenes* is uncommon as a cause of IE in HIC (44, 45). Our postulation is that there could be problems in species determination in developing countries in which any beta-hemolytic *Streptococcus* is reported as *S. pyogenes* despite that *Streptococcus dysgalactiae* and *Streptococcus agalactiae* are much more common beta-hemolytic *Streptococcus* IE. The presence of *S. viridans* recall a need for antibiotic prophylaxis among patients with structural cardiac disease such as RHD when undergoing procedures that involve gingival manipulation. The other category of microorganisms reported as a cause of IE is CoNS and enterococci. The proportion of CoNS was 7.3% in group 1 and 8.6% in group 2 while the proportion of enterococci was 6.3% in group 1 and 5.9% in group 2.

### Medical treatment

In developing countries, medical treatment is the most common treatment of IE owing to the limited availability of cardiac surgery. However, due to the unavailability of appropriate blood culture tests and susceptibility testing, empirical antibiotic therapy remains the mode of treatment. In our review, penicillin was used most frequently, with or without aminoglycoside. Of the aminoglycosides, gentamicin was the most common antibiotic used. Ceftriaxone and vancomycin were also being used regularly. Another reason for empirical antibiotic therapy in developing countries is the absence of local guidelines (informed by local data) on common microorganisms and on antibiotic resistance (46). Lastly, financial constraint is limiting access to expensive medications that may be required for antibiotic resistant bacteria (47). In developed countries, the use of partial oral treatment of IE is reported to offer early discharge out of the hospital and hence would reduce hospital complications and costs (8, 48, 49). However, this practice has not been reported in developing countries.

### Surgery

This review showed a wide range of proportions of patients who underwent surgery for IE, ranging from 0% in Lao (15) to 60.2% in China (31). The higher numbers in this range (42.3–60.2%) come from upper-middle-income countries (14, 24, 25, 27, 31). Similarly, previous studies have reported a wide variation in cardiac surgical interventions for IE in developing countries (3, 12). In contrast, in developed countries 50–75% of IE patients receive surgery (6, 8, 35, 36, 50). However, these figures may reflect a selection biased population done from tertiary centers. Indeed, studies from the Nordic countries have shown that surgery is performed in a smaller proportion of cases (51, 52). In the current review, the number of patients receiving surgery was significantly higher (38.8%) in the early cohorts compared to the late cohorts (28.8%). The reason for a smaller number of surgeries in the late cohort is that the proportion of surgeries were higher in the cohort with Letaief et al. study (24). However, in our reviewed studies we did not include complications imposed by IE but we assume that since many patients in developing countries attend late hospital, the complications are many and hence these figures are low. Indeed, two previous reviews have reported that the rates of surgery among patients with IE in developing countries are low (3, 12). There are several reasons for the low uptake of surgery for IE in developing countries. Firstly, in developing countries, there is limited access to cardiovascular surgery (22, 53, 54). There are very few countries with independent cardiac surgery programs (53), with one cardiac surgeon serving about 14 million persons in sub-Saharan Africa (55). Unlike in HIC countries like the USA where there is one cardiac center per 120,000 people, in Africa, there is one center per 33 million people (56). Secondly, even in countries where there is the availability of facilities capable of surgical interventions, the high costs of procedures are another obstacle considering that most of these patients do not have health insurance (22). Thirdly, the optimal timing for surgical intervention among patients with complications that require emergency surgery is debatable (57). Early surgery is recommended (and decreases mortality) in the setting of IE with complications such as embolic events, congestive cardiac failure, and valvular abscess (3, 35, 57–59). These complications are common among most patients with IE in developing countries because these patients are usually diagnosed late and therefore present late in the hospital. However, the observed difference among the two cohorts should be interpreted cautiously owing to a wide range of surgeries performed in the reviewed studies with Letaief et al. (24) reporting higher figures than others.

### Mortality

The current review showed that over the last four decades the in-hospital mortality imposed by IE has not significantly changed, in the early cohorts the mortality was 20.9% while in late cohorts it was 22.3%. Similarly, other studies by Njuguna et al. (3) and Noubiap et al. (12) have reported a relatively similar in-hospital mortality due to IE with an in-hospital mortality rate of 22.6% (11.2–31.2%). Surprisingly, the observed mortality is similar to the 20% that is reported in HIC (6, 35, 36). In HIC patients present early and get diagnosed early (6, 36). As could be expected, in resource-constrained countries the management of severe diseases like IE is challenging and hence mortality could be higher than that observed in HIC (1, 4, 35). There are several reasons for the observed relatively lower mortality in our review. Firstly, the most common pathogen is *Streptococcus* spp. rather than *Staphylococcus* spp. which is fatal. Secondly, could be due to the use of cardiac surgery on patients with guideline-recommended indications such as congestive heart failure. Thirdly, in developing countries patients with IE are young and with few comorbid conditions compared with patients in HIC (35, 60). It is known that old age and comorbid conditions are important predictors of increased mortality in IE patients (4).

## Strengths and limitations of the study

This review has several strengths. Firstly, we covered several decades of cohorts of IE studies (1986–2017). Secondly, we did a comparison of two cohorts to assess the trends in the changing of several parameters affecting/related to IE. Thirdly, we excluded studies that entirely recruited children to avoid skewness of our findings to one population. However, our review has several limitations. First, there was under-representation of Africa where many developing countries belongs. Second, most of the included studies were retrospective and hence subjective to all of the inherent shortcomings of retrospective studies. Fifth, many of the reviewed studies were tertiary level hospital-based and therefore the results could not be representative of the general population. Lastly, because the Letaief study comprised a very large proportion of patients in the early cohort it means that the results from this single study made a large contribution to the overall conclusions.

## Conclusions

This review is a wake-up call for addressing a scarcity of studies on IE in developing countries. Rheumatic heart disease and congenital heart disease are still the most common underlying cardiac conditions of IE. Prosthetic heart valve, DVD, intravenous drug use, and HIV are risk factors also. While the proportion of streptococci and *S. aureus* has increased, the number of NBCs and patients getting surgery has decreased over time. In the reviewed cohorts, mortality caused by IE has not changed over the past four decades.

## Recommendations

It is essential to identify the causative bacteria to offer the proper medical treatment in patients with IE. To improve outcomes of IE in developing countries, access to cardiac surgical intervention should be scaled-up. Well-designed research such as prospective cohort studies are needed and programs (such as RHD control) aiming at the reduction of morbidity and mortality caused by IE in developing countries are encouraged. A conceptual framework comprising of required baseline information (such as data on disease burden, human resources, and treatment protocols) and requirements for executing primary, secondary, and tertiary preventions has been advocated as a best model for RHD control (61). With primordial prevention and research agenda being an integral part of the program.

## Author contributions

RM and SC conceptualized the idea. RM and JV performed literature search and wrote the first draft of the manuscript. MC, PC, AK, GK, JM, AW, PK, and LF critically reviewed the manuscript. All authors contributed to the manuscript and approved the final version.

## Conflict of interest

The authors declare that the research was conducted in the absence of any commercial or financial relationships that could be construed as a potential conflict of interest.

## Publisher's note

All claims expressed in this article are solely those of the authors and do not necessarily represent those of their affiliated organizations, or those of the publisher, the editors and the reviewers. Any product that may be evaluated in this article, or claim that may be made by its manufacturer, is not guaranteed or endorsed by the publisher.

## Abbreviations

CHD: congenital heart disease
CoNS: coagulase negative *Staphylococcus*
DVD: degenerative valve disease
HIC: high income countries
HIV: human immunodeficiency virus
IE: infective endocarditis
IVDA: intravenous drug abuse
NBC: negative blood culture
PHV: prosthetic heart valve
RHD: rheumatic heart disease
USA: United States of America.
